# Case report: Initial presentation of pancreatic schwannoma as cystic pancreatic mass treated with classic Whipple’s procedure

**DOI:** 10.3389/fgstr.2024.1425831

**Published:** 2024-10-24

**Authors:** Zekewos Demissie Jemaneh, Nahom Zemedkun, Serkalem Nurlegn, Amanuel Mamuye Woldeamanuel, Henok Seife, Yohannes Birhanu, Bethelhem Berhanu Belachew

**Affiliations:** ^1^ Department of Internal Medicine, Lancet General Hospital, Addis Ababa, Ethiopia; ^2^ Division of Hepatobiliary Surgery, Department of Surgery, Addis Ababa University, Addis Ababa, Ethiopia; ^3^ Division of Gastroenterology and Hepatology, Department of Internal Medicine, Addis Ababa University, Addis Ababa, Ethiopia; ^4^ Department of Radiology, St. Paul Millennium Medical College, Addis Ababa, Ethiopia

**Keywords:** schwannoma, pancreatic tumor, pancreatic schwannoma, cystic pancreatic mass, Whipple’s procedure

## Abstract

Pancreatic schwannomas are exceedingly rare tumors arising from Schwann cells of the peripheral nerve sheath within the pancreas. Often asymptomatic or presenting with nonspecific symptoms, these tumors pose a diagnostic challenge due to their mimicry of other pancreatic neoplasms on imaging studies. Histologically, pancreatic schwannomas demonstrate spindle cell proliferation with a distinct Immunohistochemical profile, including positive staining for S-100 protein. Surgical resection remains the cornerstone of treatment, with excellent long-term prognosis following complete excision. Here, we present a case report of a pancreatic schwannoma in a woman presenting with a cystic pancreatic mass, underscoring the importance of considering this rare entity in the differential diagnosis of pancreatic lesions.

## Introduction

Pancreatic schwannomas represent an exceptionally rare subset of pancreatic tumors, with fewer than 50 cases documented worldwide (1, 2). Originating from Schwann cells of the neural sheath, these tumors are predominantly benign and exhibit slow growth kinetics (1). Notably, the head of the pancreas is the most commonly affected site, accounting for approximately 40% of cases (2, 3). While typically benign, malignant transformations can occur, particularly in association with increased tumor size or in patients with underlying conditions such as neurofibromatosis type 1 or von Recklinghausen’s disease (4, 5). Degenerative changes, including cystic formation, calcification, and hemorrhage, are observed in around two-thirds of cases, underscoring the diverse histopathology features of pancreatic schwannomas (1, 6). Given their potential for varied clinical presentations, variable imaging features and histological characteristics, accurate preoperative diagnosis, even though difficult, is paramount for determining appropriate management strategies. Surgical resection remains the cornerstone of treatment, offering a curative approach for most patients afflicted with this rare pancreatic neoplasm (2, 3).

## Case presentation

We present a case of a 60-year-old previously healthy female who sought medical attention at our outpatient clinic due to persistent abdominal discomfort lasting for four months, with recent exacerbation over three weeks. The pain was described as non-specific, without any associated symptoms such as nausea, vomiting, or changes in bowel habits. Upon physical examination, there were no palpable masses, tenderness, or signs of organomegaly. Laboratory investigations, including complete blood count, liver function tests, renal function tests, and urine and stool analysis, revealed no abnormalities.

Given the persistence of symptoms and the need to rule out underlying pathology, imaging studies were conducted. A contrast-enhanced CT scan of the abdomen revealed a well-defined cystic mass measuring 3.2 by 2.8 cm located in the uncinate process of the pancreas. Further characterization of the lesion was achieved through MRI, which demonstrated T1 hypo-intensity and T2 hyper-intensity, consistent with a cystic lesion.

**Figure 1:**
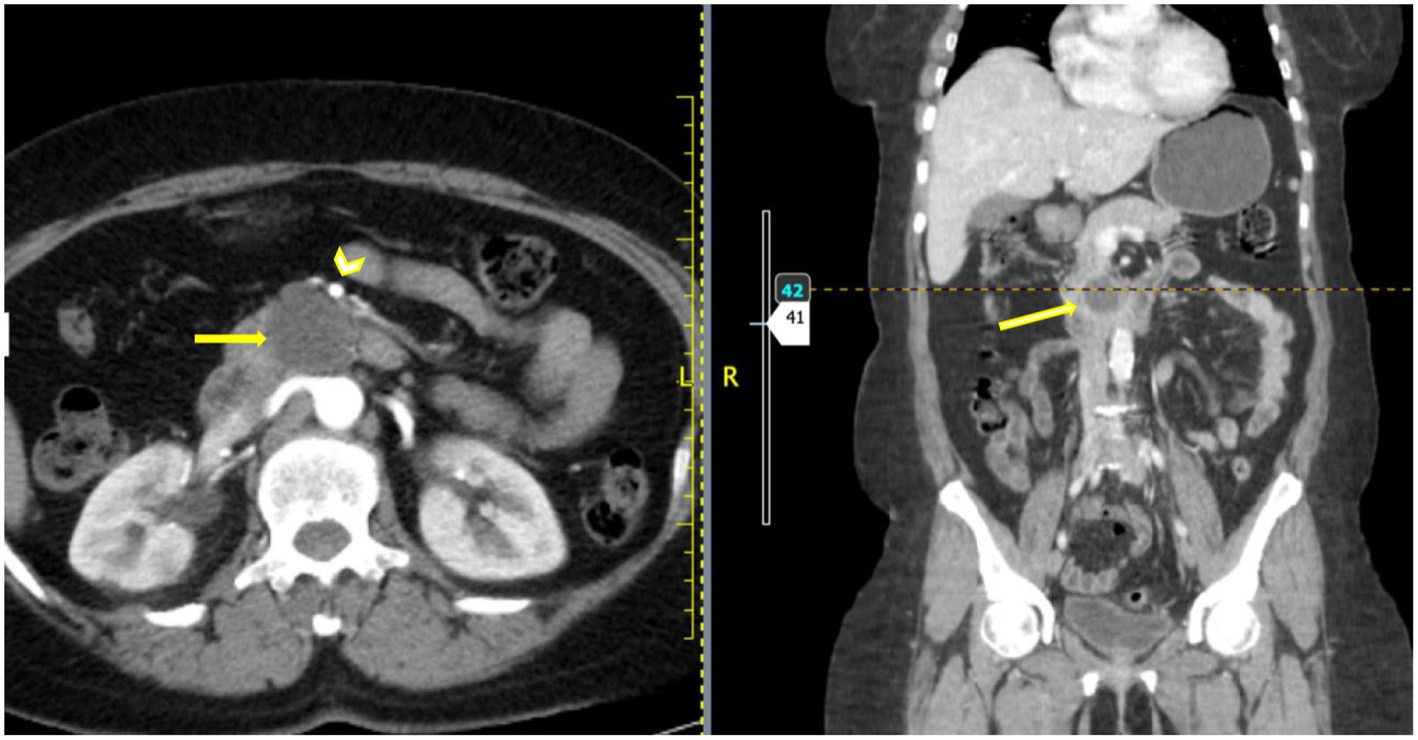
Axial and coronal post contrast CT images showing a hypo-enhancing well defined rounded mass (yellow arrow) which appears to be clawed from the uncinate process of the pancreas. The SMA is seen anterior to the mass (arrowhead) with a clear fat plane from the mass.

The CT scan images are shown in **Figure 1** below.

Considering the potential malignant nature of the mass, patient underwent a classic Whipple pancreaticoduodenectomy procedure two days following the imaging studies. Intraoperative, a cystic mass was palpated on the uncinate process of the pancreas, with no evidence of invasion into adjacent structures or metastasis to regional lymph nodes. Biopsy specimens were obtained and subjected for histopathology which suggested pancreatic schwannoma and Immunohistochemical staining for S-100 protein confirmed the diagnosis.

Postoperatively, the patient recovered well without any complications and was discharged home in stable condition. Regular follow-up visits were scheduled in the outpatient clinic. A repeat CT scan performed two weeks postoperatively showed no evidence of residual or recurrent disease.

## Discussion

Mesenchymal tumors, constituting approximately 1%–2% of all pancreatic tumors, are classified based on their cell of origin. Among these, schwannomas, also referred to as neurilemomas, are benign spindle cell tumors derived from Schwann cells lining nerve sheaths. They commonly occur in various anatomical locations, with the lower extremity being the most prevalent, followed by the upper extremity, trunk, head and neck, retroperitoneum, mediastinum, and pelvis (7–9).

Pancreatic schwannoma, an exceptionally rare occurrence, accounts for less than 1% of all schwannomas of which 2/3^rd^ of the tumors exhibit cystic characteristics (6). These tumors originate from autonomic sympathetic and parasympathetic nerve fibers, or from branches of the vagus nerve that extend to the pancreas. In terms of their location within the pancreas, pancreatic schwannomas are predominantly found in the head (40%), followed by the corpus (21%), neck (6%), tail (15%), and uncinate process (13%), respectively (1, 3).

Due to their slow growth, schwannomas are prone to degenerative changes such as hemorrhage, cyst formation, necrosis, and calcification. These degenerative schwannomas can sometimes resemble pancreatic cystic neoplasms, neuroendocrine tumors, cystadenoma, cystadenocarcinoma, and pancreatic pseudocysts (1, 10, 11).

Even though pancreatic schwannomas usually possess benign nature, there are factors that can increase the likelihood of malignant transformation. These include larger tumor size, high mitotic activity, genetic mutations such as Ki-67, p53, and Bcl-2 (4, 5, 12).

The symptoms of patients with pancreatic schwannoma vary. According to the study conducted by Moriya et al., abdominal pain was the most commonly reported symptom. Additionally, 30% of the patients were asymptomatic. Other reported symptoms include weight loss, back pain, nausea and vomiting, melena, and jaundice were among the other observed symptoms and signs. This study couldn’t correlate symptomatology of patients with the size as well as location of the tumor (1, 3).

The need for imaging is crucial for preoperative diagnosis of pancreatic schwannomas since the majority of patients show no symptoms. On computed tomography (CT) scan, these tumors characteristically exhibit of low-density areas or cystic images (3, 13). While CT and MR findings do not reliably differentiate between benign and malignant lesions, MRI typically depicts schwannomas as hypointense on T1-weighted images and hyperintense on T2-weighted images. Despite these characteristic features, confusion with other pancreatic tumors is common, necessitating careful consideration in the differential diagnosis. Despite the wide unavailability of Endoscopic Ultrasounds, Ultrasound-guided Fine Needle Aspiration (EUS-FNA) biopsy is becoming more prevalent in clinical practice, offering potential for accurate preoperative diagnosis, addressing the conundrum of distinguishing schwannomas from other tumors. Key features in endoscopic ultrasound indicative of schwannoma are round, well-demarked, solid homogeneous and hypoechoic mass, with hypo-enhancing and avascular structures in contrast-enhanced harmonic EUS (13–18).

Immunohistochemical staining is often necessary for precise diagnosis. It strongly stains positively for S-100 protein. Further laboratory investigations, including CA 19-9 and CEA levels, serve as valuable tools in ruling out potential differentials like pancreatic adenocarcinoma. Typically, their values either remain undetectable or fall within normal ranges in patients with this tumor (16).

Enucleation is highly effective treatment option, particularly notable due to the encapsulated and typically benign nature of these tumors, with the anterior approach preventing hemorrhages (19). The posterior approach is preferred for intrasacral schwannomas. Incomplete resections carry a 10% recurrence risk, necessitating further follow-up (20, 21). For peripheral schwannomas, open or laparoscopic surgery, particularly the anterior approach for retroperitoneal schwannomas, is recommended (20). The Whipple procedure or distal pancreatectomy may be considered for suspected malignant transformation. Moriya et al. (3) reported pancreaticoduodenectomy in 32% of cases, distal pancreatectomy in 23%, enucleation in 14%, and resection in 4%, with some patients deemed unfit for surgery.

The prognosis for patients with pancreatic schwannoma after successful surgery is generally favorable, with a high likelihood of cure and long-term survival. Schwannomas is associated with excellent long-term prognosis and minimal risk of disease recurrence or metastasis (3, 22).

## Conclusion

Pancreatic schwannomas are rare tumors that should be considered in the diagnosis of pancreatic cystic tumors. Surgical resection is the preferred treatment, with ongoing follow-up being standard practice. Diagnosis before surgery is challenging, but certain imaging features can raise suspicion. Enucleation of the tumor is recommended if feasible, though more extensive resection may be needed in some cases. Overall, based on current literature, virtually all patients appear to achieve cure regardless of the surgical approach.

## Data Availability

The original contributions presented in the study are included in the article/supplementary material. Further inquiries can be directed to the corresponding author.
